# Emotional response evoked by viewing facial expression pictures leads to higher temporal resolution

**DOI:** 10.1177/20416695231152144

**Published:** 2023-02-09

**Authors:** Misa Kobayashi, Makoto Ichikawa

**Affiliations:** Graduate School of Science and Engineering, 12737 Chiba University, Chiba, Japan; 53347 Japan Society for the Promotion of Science, Chiyoda-ku, Japan; Department of Psychology, 12737 Chiba University, Chiba, Japan

**Keywords:** arousal, valence, method of constant stimuli, facial inversion effect

## Abstract

We examined the effects of emotional response, with different levels of valence and arousal, on the temporal resolution of visual processing by using photos of various facial expressions. As an index of the temporal resolution of visual processing, we measured the minimum lengths of the noticeable durations for desaturated photographs using the method of constant stimuli by switching colorful facial expression photographs to desaturated versions of the same photographs. Experiments 1 and 2 used facial photographs that evoke various degrees of arousal and valence. Those photographs were prepared not only in an upright orientation but also in an inverted orientation to reduce emotional response without changing the photographs’ image properties. Results showed that the minimum duration to notice monochrome photographs for anger, fear, and joy was shorter than that for a neutral face when viewing upright face photographs but not when viewing inverted face photographs. For Experiment 3, we used facial expression photographs to evoke various degrees of arousal. Results showed that the temporal resolution of visual processing increased with the degree of arousal. These results suggest that the arousal of emotional responses evoked by viewing facial expressions might increase the temporal resolution of visual processing.

Anecdotally, it is often described that the effects of strong frightening emotions cause subjective time to slow during life threatening events such as car accidents. Emotions have been shown to affect subjective time. For instance, an individual who is frightened by an object within the visual field tends to overestimate the duration of the object's presence (e.g., Angrilli et al., 1997; Droit-Volet et al., 2004; Watts & Sharrock, 1984).

If one sees events develop in slow motion when feeling strong emotions, then one might detect a brief event more easily than when seeing those things develop as usual with a normal mental state. Stetson et al. (2007) investigated this very point. They examined how emotion affects the temporal resolution of visual processing by evoking fear with a 31-m free fall. They found no evidence of increased temporal resolution during free fall, although their participants retrospectively estimated their own fall as lasting 36% longer than others’ falls. Based on these findings, Stetson et al. (2007) proposed duration dilation during a frightening event and claimed that a lack of concomitant increase in temporal resolution would be caused not by improvement of the actual temporal resolution of visual processing but rather retrospectively by the richer encoding of memory during the events.

Nevertheless, as described by Stetson et al. (2007), because their display was not fixed in the visual field and because measurements were taken for each participant only during a single fall (about 2.5 s), their experimental methods led to some difficulties in measuring the accurate temporal threshold for visual perception with each emotional state. These difficulties reported in a study by Stetson et al. (2007) make it difficult to ascertain whether strong emotions have any effect on the temporal resolution that is associated with visual processing.

In our earlier study (Kobayashi & Ichikawa, 2016), we examined the effects of emotion on the temporal resolution of visual processing with lab experiments using fear-inducing and safe images selected from the International Affective Picture System (IAPS; Lang et al., 2008). As an index of the temporal resolution associated with visual processing, we switched a colorful emotional image to monochrome versions of the same image after 1 s presentation. Then we measured the duration of the period when the monochrome image was noticeable. Results showed that the minimum duration when an observer was able to notice the monochrome image presentation when viewing a fearsome image was shorter than when viewing non-fearsome images. Additionally, results indicate that the frequency with which participants correctly reported a monochrome image was significantly and positively correlated with the emotional arousal scores of the presented images. These results suggest that the emotion of fright might increase the temporal resolution of visual processing.

Although the methodology described by Kobayashi and Ichikawa (2016) prepared a better environment for accurate measurement of the temporal resolution associated with visual processing than an earlier study (Stetson et al., 2007) by overcoming several difficulties pointed out for the study, it presents other potential hurdles. Because images from IAPS were selected from natural images with various everyday objects and scenes, the properties of the images used for the study by Kobayashi and Ichikawa (2016) (such as profiles related to luminance, colors, and spatial frequencies) might vary among emotional conditions. Although no correlation was found between those stimulus features and the results of our earlier study, such properties of images might affect the temporal resolution of visual processing in our earlier study (Nomura & Yotsumoto, 2018). To evaluate the effects of some emotions evoked by viewing images, the effects of these properties of images must be controlled.

For this study, we used images of facial expressions to evoke different emotions to investigate some emotional effects on the temporal resolution of visual processing without changing the properties of the images among the emotional conditions. Compared to the scenery images used for our earlier study, the variance of image features related to luminance, colors, and spatial frequencies among the facial images of a single person with different expressions was expected to be small. More importantly, we prepared the control condition by inverting the same facial images that we used to evoke different emotions. Inverting the facial images presumably reduces the evoked emotion without changing any property of the facial images in terms of the facial inversion effects. Humans reportedly have greater difficulty perceiving inverted faces than upright faces (e.g., Thompson, 1980; Yin, 1969). Earlier studies have demonstrated that presenting images of facial expressions is effective for modulating the subjective duration by evoking an emotional response (e.g., Droit-Volet et al., 2004).

Kobayashi and Ichikawa (2016) revealed that the effects of emotion on temporal resolution in visual processing do not correlate with the effects of emotion on perceived duration. For this study, therefore, we specifically examined the effects of emotion on the temporal resolution of visual processing.

Bocanegra and Zeelenberg (2011) used facial expression images, specifically fearful and neutral facial expressions, to assess the effects of emotion on the temporal resolution of visual processing at a suprathreshold level for temporal discontinuity detection. They reported that viewing a fearful face improves the temporal resolution of visual processing, but that it impairs the spatial resolution of visual processing. In their temporal resolution experiment (Experiment 1), 100 ms after presenting fearful faces or neutral faces on both the left and right sides of a fixation point, they presented two consecutive Landolt circles at either side of the fixation point with different intervals of 10–30 ms in 50% of the trials. In the other 50% of trials, they presented a single Landolt circle. Their participants were asked to judge whether the Landolt circle was flickering in each trial. They analyzed the performance in terms of the signal detection theory measure *d′* for each condition, which revealed that *d′* increased from about 3.0 for the neutral face to about 3.3 for the fearful face, whereas the hit rate increased from 0.884 for the neutral face to 0.921 for the fearful face. Their results suggest that viewing a fearful face might increase the temporal resolution of visual processing at a suprathreshold level for the temporal discontinuity detection task. Actually, those results are expected to be compatible with an increased temporal resolution of visual processing at a near-threshold level, as suggested by Kobayashi and Ichikawa (2016). To assess the importance of the information provided to the amygdala, Bocanegra and Zeelenberg (2011) presented facial expression images that were restricted to low-spatial-frequency components or that were restricted to high-spatial frequency components (Experiment 2). They found enhanced performance in their temporal resolution task with images having low-spatial frequency; *d′* increased from about 2.7 for the neutral face to about 3.1 for the fearful face presented. Additionally, they demonstrated that such increased performance for their temporal resolution task in terms of viewing a fearful face ceased to occur if the low-spatial frequency component of the facial images was presented with an inverted (upside down) orientation (Experiment 3); *d′* remained at about 2.5 for the neutral face and at about 2.3 for the fearful face. Although Bocanegra and Zeelenberg (2011) examined the effects of emotional expressions on the temporal resolution of visual processing at a suprathreshold level, their results suggest that inversion of facial expressions is useful to assess some effects of emotional faces at a near-threshold level without changing the properties of facial images.

For this study, we examined whether the emotional response evoked by viewing facial images increases the temporal resolution of visual processing at a near-threshold level, as suggested by Kobayashi and Ichikawa (2016). To assess the effects of arousal and valence emotional responses, we used various facial expressions for Experiment 1, whereas Bocanegra and Zeelenberg (2011) used only a fearful face as an example of emotional expression. For Experiment 1, we presented negative (fearful and angry) and positive (joyful) expressions as well as neutral faces before presenting stimuli for temporal resolution measurements. For Experiment 2, we presented the same stimuli, but inverted, to reduce the emotional response that is felt when viewing the facial expression images. For Experiment 3, we presented angry, sad, and neutral facial expressions, which were expected to evoke high, medium, and low arousal, respectively (Russell & Mehrabian, 1977). Based on the results obtained from these three experiments, we discuss how emotional arousal and valence affect the temporal resolution of visual processing.

## Experiment 1

For Experiment 1, after using various facial expressions to evoke different emotions, we examined how arousal and valence affect the temporal resolution of visual processing. We prepared fearful, angry, joyful, and neutral faces as stimuli. Both fearful and angry faces were expected to evoke high arousal and low (negative) valence as emotional responses. The joyful face was expected to evoke high arousal and high (positive) valence. A neutral face was expected to evoke low arousal and neutral valence. After measurement of the temporal resolution of visual processing, observers rated the evoked emotions, the arousal that they had experienced, and the valence while viewing each facial expression image. Based on those ratings, we confirmed whether the expected emotion had been evoked.

### Methods

#### Observers

Observers were 19 naïve students: 10 women and 9 men (*M* and *SD* of age were respectively 21.47 and 2.62 years). All had normal or corrected-to-normal visual acuity. Before the experiments, observers provided written informed consent to participate. Particularly, they were advised that some of the images might be considered shocking. They were advised that they would be able to stop the presentation and quit the experiment at any time. No participant requested that the experimenter stop the experiment. All observers finished the observations with no difficulty.

#### Apparatus

A personal computer (Vostro 420; Dell Inc.) presented stimuli on a 17-in. display (100 Hz, T561; Eizo Corp.). The viewing distance was about 57 cm. Each observer sat on a chair in front of a desk (80 cm height), with the observer's head fixed on a chin rest. Stimulus presentation and recording of observer responses were conducted using software (Super Lab ver. 4.5; Cedrus Corp.).

#### Stimuli

We used anger, fear, joy, and neutral facial expressions of two Asian women and two Asian men selected from photographs of the Advanced Telecommunications Research Insutitute International (ATR) facial expressions database DB99 (ATR Promotions, 2006). We conducted a preliminary test to select facial images from the database that would be regarded as angry, fearful, and joyful expressions. The facial image size was fixed at 7.0 ×  4.0°. The display background was black (0.005 cd m^−2^). We prepared 70% desaturated images from the 16 original images using an image editing program (Adobe Photoshop CS5 Extended ver. 12.0.4; Adobe Inc.).

#### Procedure

**Figure 1.**
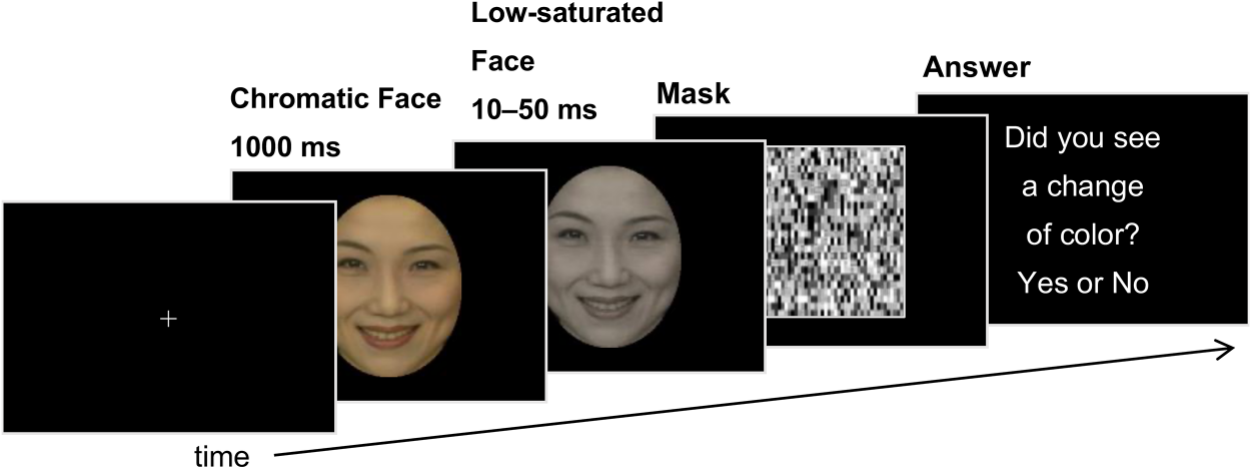
Trial format for Experiment 1.

A white cross (0.7 ×  0.7°) was presented at the center of the display (Figure 1) for fixation. The observer pressed a space key to start each trial with the fixation on the center cross. At 1,000 ms after the key press, one facial expression image appeared for 1,000 ms. In the five-sevenths of the trials, the colored image was followed by a desaturated version of the same image. Five duration conditions were used for the desaturated image: 10–50 ms by 10 ms steps. In the other two-sevenths of the trials, the original color images were presented with no disruption for the same duration as that used for the desaturated trials. Then the display turned to a random dot mask. Observers reported whether they saw the desaturated image, or not. Each of the 16 facial images was presented five times with five presentation duration conditions for desaturated images (400 trials) and twice with five presentation duration conditions for catch trials (160 trials) in random order. Therefore, each observer completed 560 trials.

After finishing the temporal resolution task, all observers assigned ratings to each image. Each image was presented for 1,000 ms. Observers rated the extent of anger, fear, and joy observed for the images using an 11-point scale (0–100) and used a bipolar seven-point scale, where −3 is the most relaxed/depressed and +3 is the most excited/pleasant, to report the arousal and valence that a facial expression made them feel while viewing the images.

### Results and Discussion

The rated values of the expressed facial emotions were always the highest among the four facial expressions (Table A1). We conducted a one-way repeated measures analysis of variance (ANOVA) with the facial expression as a factor for the arousal and valence ratings. The main effect of the facial expressions was significant for the arousal rating [*F*(3, 54) = 93.83, η_*p*_^2^ = 0.84, *p *< .0001] and for the valance rating [*F*(3, 54) = 82.48, η_*p*_^2^ = 0.82, *p *< .0001]. Shaffer's post hoc tests with Bonferroni correction revealed that the arousal ratings for the anger, fear, and joy conditions were higher than those for the neutral condition. The tests also showed that the valence ratings for the joy condition were higher than those of the other three conditions, whereas valence ratings for the joy and neutral conditions were higher than the anger and fear conditions (*p *< .0001). These results corresponded to our expectations (Russell & Mehrabian, 1977) for the expressions used for this study.

**Figure 2.**
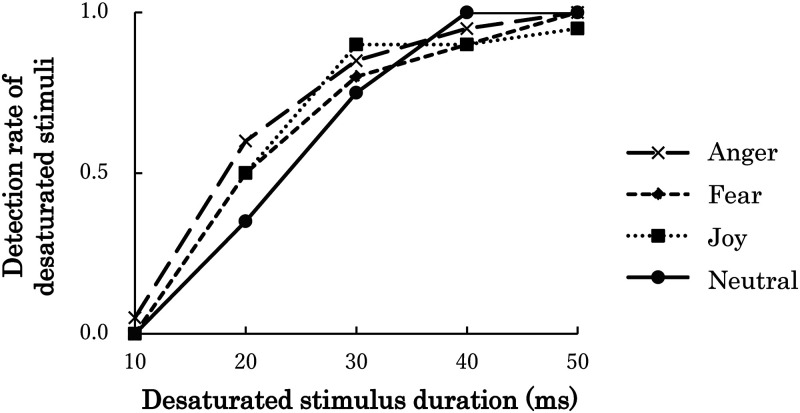
Detection rates of desaturated stimuli in each stimulus duration for one participant in Experiment 1.

We excluded the data of four observers for whom the false alarm (FA) rate exceeded 50% in any condition. The mean FA rate of the remaining 15 observers was 2.0% (*SD* = 0.02). Figure 2 presents the frequency of correct detection of desaturated images in each duration condition for one observer.

**Figure 3.**
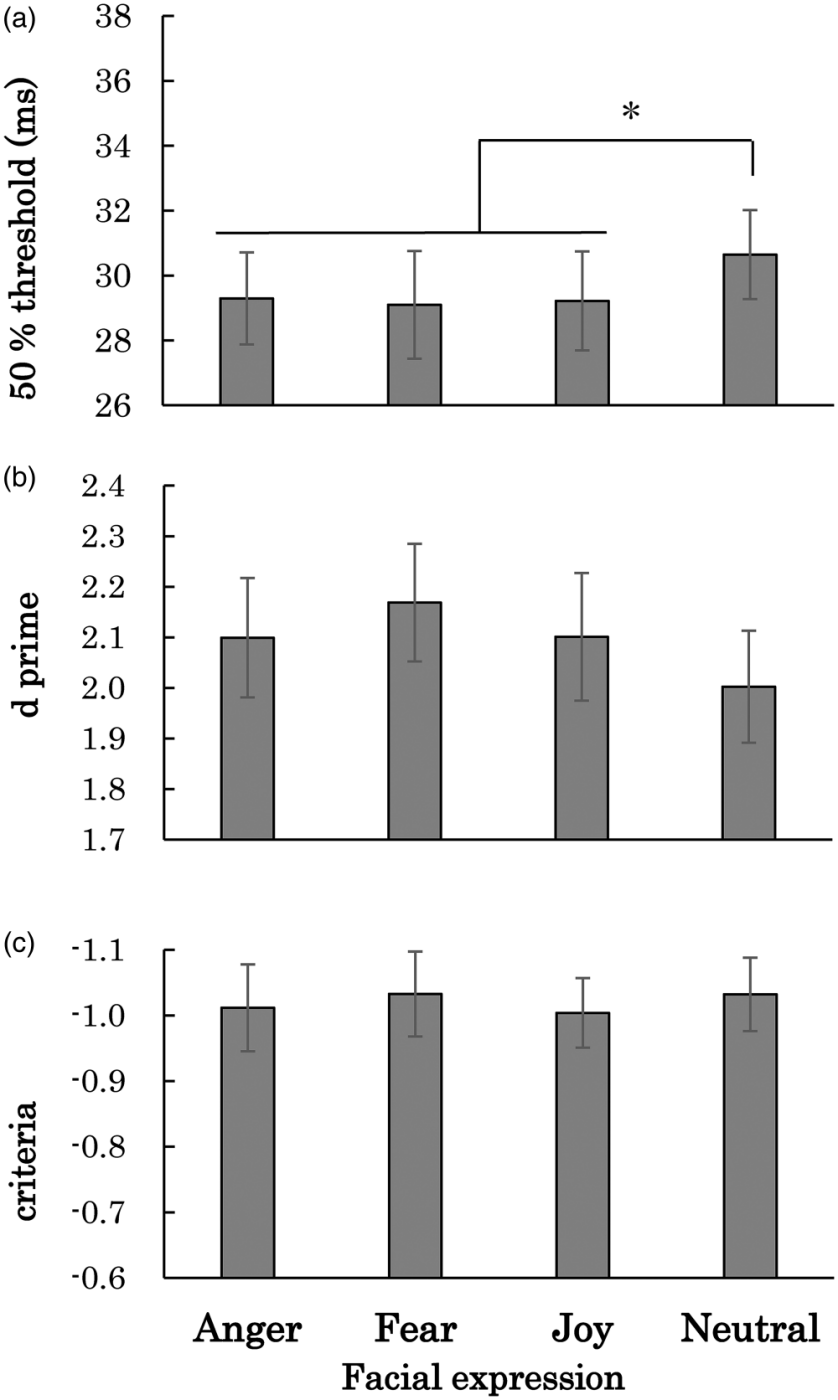
(a) Average of the 50% thresholds for the detection of desaturation for each facial expression condition in Experiment 1. (b) Average of *d*′ for each facial expression condition. (c) Average of the criteria for facial expression conditions. Error bars show SEM. *Note.* * means *p *< .05.

We performed a Probit analysis (Finney, 1971) to obtain individual 50% thresholds of the detection of desaturation for each condition. Figure 3a shows the means of 50% thresholds for the respective facial expression conditions from 15 participants whose data fitting would be good (*p *> .05) in all the expression conditions in χ^2^ tests. The means of the upper-lower limits of the 95% confidence intervals with the estimated 50% thresholds for the anger, fear, joy, and neutral conditions were 24.22–34.00 ms, 22.85–35.18 ms, 21.25–37.41 ms, and 26.38–34.77 ms, respectively. One-way repeated measures ANOVA with the facial expression as a factor for the 50% thresholds of detection of desaturation from these 15 participants revealed a significant main effect of the expressions [*F*(3, 42) = 4.14, η_*p*_^2^ = 0.23, *p* = .01]. Shaffer's post hoc tests with Bonferroni correction revealed that the 50% thresholds for the detection of desaturation for the anger, fear, and joy conditions were significantly lower than for the neutral condition (*p* = .01), although no significant difference was found among the anger, fear, and joy conditions despite the valence difference.

To examine how criteria difference is involved in the differences in desaturation detection among facial expression conditions, we conducted one-way repeated measures ANOVA with the facial expression as a factor for the *d′* (Figure 3b) and criteria (Figure 3c). Neither the analysis for the *d′* nor the criteria indicated a significant main effect of facial expression [*F*(3, 42) = 1.08, η_*p*_^2^ = 0.07, *p* = .37 for *d′* and *F*(3, 42) = 0.19, η_*p*_^2^ = 0.01, *p* = .90 for criteria]. These results show that the difference of 50% threshold with facial expression cannot be attributed to the difference of criteria in detecting desaturation of the pictures. Bocanegra and Zeelenberg (2011) found a significant increase in *d′* when viewing some facial expressions (such as fearful face), although we did not find such an increase in *d′* with facial expressions. This difference for *d′* between the present and their studies is expected to depend on differences in the detection task level: we use the near-threshold level task, whereas Bocanegra and Zeelenberg (2011) used the suprathreshold level task (hit rates in our Experiment 1 were, at most, 0.552 for joyful faces, whereas those in their study were 0.921 for the fearful faces and 0.884 for the neutral faces). In addition, the differences in procedures between these studies might cause the differences: in our study, the observer's task was to detect the desaturation in the facial images, which were continuously presented to evoke an emotional response, whereas the task in Bocanegra and Zeelenberg (2011) was to detect the temporal gap between two consecutive Landolt circles, which were presented immediately after the facial images. In our study, in which observers tried to detect the desaturation in the continuously presented facial images, both the hit rate and the FA rate would remain at low levels because of the visual persistence compared with their methods. Low levels of hit rate and FA rate would produce a small *d′* in our study. In fact, *d′* in Bocanegra and Zeelenberg (2011) were greater than 3.0, whereas it was around 2.0 in our study. One may have more difficulty in finding significant differences among conditions for small *d′* than for large one.

Because the sample used for the present experiment was on the small side, we calculated the Bayes factors (Lee & Wagenmakers, 2014) using Bayesian repeated measures ANOVA in terms of JASP (JASP Team, 2022) to confirm the difference in 50% thresholds obtained from Probit analyses between the neutral face and the faces with anger, fear, and joy expressions. One-way Bayesian repeated measures ANOVA with the facial expressions as a factor for the 50% thresholds for the detection of desaturation revealed a Bayes factor (BF_10_) of 4.124; post hoc tests revealed BF_10_ of 4,172, 4.956, and 9.899, respectively, for the differences between the neutral face and the anger, fear, and joy conditions. These values of the Bayes factors are within the level of the moderate evidence for H_1_ (Keysers et al., 2020). The obtained data were more likely under the alternative hypothesis (emotion evoked by viewing facial expressions might increase the temporal resolution) than under the null hypothesis. One-way Bayesian repeated measures ANOVA with the facial expressions as a factor for the *d′* and criteria revealed Bayes factors (BF_10_) of 0.267 and 0.095, respectively. These values of the Bayes factors provide evidence in favor of the null hypothesis that no difference exists in performance between these facial expressions.

Viewing the facial expressions that would evoke emotions such as anger, fear, and joy enhanced the temporal resolutions more than the neutral expression did. No significant difference was found between the temporal resolutions reported after viewing the positive-valence expressions (joy) and after viewing the negative-valence expressions (fear and anger). These results suggest that the minimum duration for detecting the desaturated facial images with salient emotion would be shorter than that of the images of neutral faces, irrespective of the evoked emotions. However, the correlation between the arousal rating for each picture and the 50% threshold for the detection of desaturation inferred from Probit analysis was marginally significant [*r* = −.466, *t*(14) = −1.97, *p* = .0687], whereas the correlation inferred between the valence rating for each picture and 50% threshold was not significant [*r* = .09, *t*(14) = 0.34, *p* = .7414]. In addition, no correlation was significant between *d′* and the arousal rating [*r* = .28, *t*(14) = 1.11, *p* = .2904] or valence rating [*r* = −.21, *t*(14) = −0.82, *p* = .4245] for each picture.

In our earlier study (Kobayashi & Ichikawa, 2016), in which we used natural images from IAPS to evoke emotions, we found a positive correlation between the rated arousal level for individual images and the obtained temporal resolution of the visual processing of those images. In that study, the arousal level of the negative stimuli (mean = 6.64, *SD* = 0.37) was slightly higher than that of the positive stimuli (mean = 4.49, *SD* = 0.77), when measured using a nine-point scale of 1–9 (Lang et al., 2008). The present results indicate that the arousal rating for individual pictures and the 50% threshold at which the detection of desaturation was marginally significant would result from large variation in the arousal rating for angry (mean = 2.54, *SD* = 0.83), fearful (mean = 1.68, *SD* = 1.71), neutral (mean = −1.84, *SD* = 1.73), and especially joyful (mean = 0.33, *SD* = 2.44) faces, with a seven-point scale of −3 to 3, compared with the variation found in our earlier study (*SD* was 0.77 at most). Experiment 3 would specifically examine this issue using facial expressions with a smaller variation in the arousal rating than the variation in Experiment 1.

## Experiment 2

When facial stimuli are presented in an inverted orientation, a person has difficulty perceiving the correct facial expression (e.g., Thompson, 1980). For Experiment 2, we presented the same facial images as in Experiment 1, but they were presented upside down without changing any image feature of the stimulus. If any image feature was responsible for raising the resolution of visual processing in Experiment 1, then one might find similar effects for the inverted facial images. However, if perceiving the facial expressions were responsible for raising the resolution of visual processing, we would find little improvement of the temporal resolution of visual processing for the inverted facial images. As in Experiment 1, after measuring the temporal resolution of visual processing, observers rated the evoked emotion while viewing each facial expression image.

### Methods

#### Observers

For this experiment, observers were 18 naïve students: 11 women and 7 men (*M* and *SD* of age were, respectively, 21.72 and 1.79 years). Eight of them participated in Experiment 1. All had normal or corrected-to-normal visual acuity. Before experiments, observers provided written informed consent as described in Experiment 1.

#### Stimuli

We used the same anger, fear, joy, and neutral facial expressions in two Asian female photographs and two Asian male photographs as those used in Experiment 1 with an inverted (upside down) orientation. As in Experiment 1, we prepared 70% desaturated images from the 16 original images.

#### Procedure

The same procedures as those used for Experiment 1 were executed, except that the facial stimuli were presented with an inverted orientation. In addition, there were six duration conditions for the desaturated image of 10–60 ms by 10 ms steps because 10–50 ms seemed insufficient for inverted faces in preliminary observation. Each of the 16 images was presented five times with six presentation duration conditions for desaturated images (480 trials) and twice with six presentation duration conditions for catch trials (192 trials). Therefore, each observer completed 672 trials.

After finishing the temporal resolution task, observers assigned ratings to each image. Each image was presented for 1,000 ms in an inverted orientation. Observers rated the extent of anger, fear, and joy using an 11-point scale (0–100%) and used a bipolar seven-point scale to report the arousal and valence experienced when viewing the images in an inverted orientation.

### Results and Discussion

The rated values of the expressed facial emotions were always the highest among the four facial expressions (Table A2). We conducted one-way repeated measures ANOVA with the facial expression as a factor for the arousal and valence ratings. The main effect of the facial expressions was not significant for the arousal rating [*F*(3, 54) = 0.42, η_*p*_^2^ = 0.02, *p* = .7413], but it was significant for the valance rating [*F*(3, 54) = 5.04, η_*p*_^2^ = 0.22, *p* = .0038]. Shaffer's post hoc tests with Bonferroni correction revealed that the valence ratings for the angry condition were higher than those for the neutral and joyful conditions (*p* = .0387, *p* = .0371). These results suggest that the emotional responses evoked by viewing the facial expressions in an inverted orientation were less stable than those evoked by viewing them in an upright orientation.

The mean FA rate of all the observers was 2.1% (*SD* = 0.03). The FA rate of no observer exceeded 50% in any condition.

**Figure 4.**
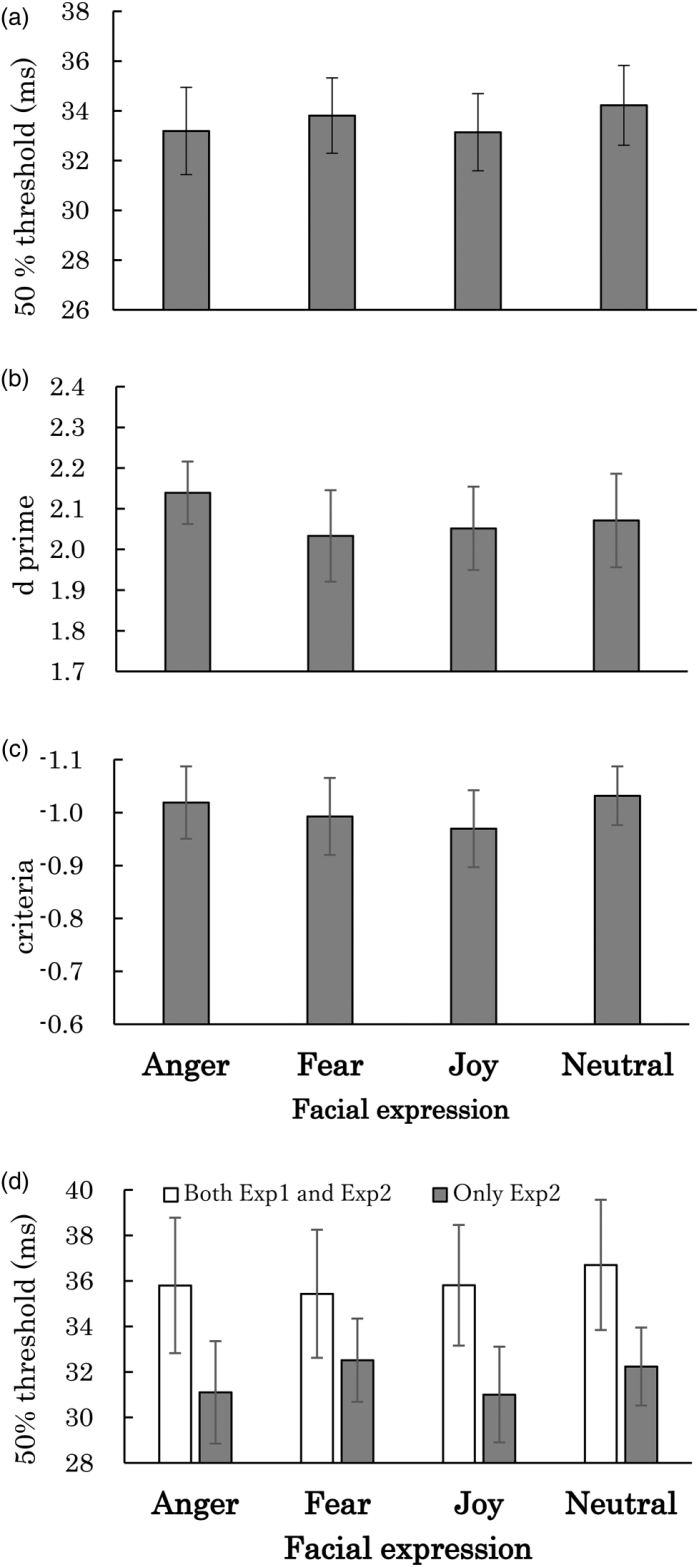
(a) Average of the 50% thresholds for the detection of desaturation for each facial expression condition in Experiment 2. (b) Average of *d*′ for each facial expression condition. (c) Average of the criteria for facial expression conditions. (d) Average of the 50% thresholds for the detection of desaturation for each facial expression condition and participant group who took part in both Experiments 1 and 2 (white bar) and that who took part only in Experiment 2 (gray bar). Error bars show SEM.

We performed a Probit analysis to obtain individual 50% thresholds for the detection of desaturation for each condition. Figure 4 presents the means of 50% threshold for each facial expression condition from 18 participants whose data fitting would be good (*p *> .05) in all the expression conditions in χ^2^ tests. The means of the upper-lower limits of the 95% confidence intervals with the estimated 50% thresholds for the anger, fear, joy, and neutral conditions were 27.35–38.14 ms, 28.47–38.90 ms, 27.58–38.48 ms, and 28.57–39.62 ms, respectively. One-way repeated measures ANOVA indicated no significant main effect of facial expressions [*F*(3, 51) = 1.21, η_*p*_^2^ = 0.07, *p* = .3171]. Because the properties of images used in Experiment 2 were the same as those used in Experiment 1, the results of Experiment 2 suggest that the emotion evoked by viewing facial expressions, not the properties of images of each facial expression condition, is responsible for the increased temporal resolution of the visual processing observed in Experiment 1.

We conducted one-way Bayesian repeated measures ANOVA with the facial expression as a factor for the 50% thresholds for the detection of desaturation. It revealed Bayes factor (BF_10_) of 3.846, which is within the level of the moderate evidence for the alternative hypothesis. Post hoc tests revealed BF_10_ of 0.588, 0.276, and 0.518, respectively, for the differences between the neutral face and the anger, fear, and joy conditions. These values of the Bayes factors provide evidence in favor of the null hypothesis that no difference exists in performance between these facial expressions. These results suggest that, although the 50% thresholds for the detection of desaturation might vary with the facial expression conditions, the difference in the 50% thresholds between the neutral face and the other three facial conditions in Experiment 1 cannot result from the properties of the images of each facial expression condition.

We conducted one-way repeated measures ANOVA with the facial expression as a factor for the *d′* and criteria. The analysis for the *d′* and criteria found no significant main effect of the emotion [*F*(3, 51) = 0.51, η_*p*_^2^ = 0.03, *p* = .6747 for *d′* and *F*(3, 51) = 0.78, η_*p*_^2^ = 0.04, *p* = .5130 for criteria]. These results indicate that there was no significant effect of facial expression on the *d′* or criteria. One-way Bayesian repeated measures ANOVA with the facial expressions as a factor for the *d′* and criteria revealed Bayes factors (BF_10_) of 0.127 and 0.166, respectively. These values of the Bayes factors provide evidence in favor of the null hypothesis that no difference exists in performance between these facial expressions.

One may suspect that the null results for the inverted faces in Experiment 2 were caused by the practice effect because eight participants took part in Experiment 1 before Experiment 2; repeat observation of specific faces with some expression might reduce the emotional response in viewing anger, fear, and joyful faces (Ishai et al., 2004). In such a case, the 50% thresholds for these faces would be elevated to the same level with that of the neutral face. We conducted ANOVA with the facial expression and participant group (eight participants who took part in both Experiments 1 and 2, and ten participants who took part only in Experiment 2) as factors for the 50% threshold obtained in Experiment 2 (Figure 3d). Although mean thresholds from the participants who took part in both experiments were larger than those from the participants who took part only in Experiment 2, the main effect of the participant group was not significant (*F*(1, 16) = 1.70, η*p*^2^ = 0.10, *p* = .21). Also, the main effects of facial expression (*F*(3, 48) = 0.92, η_*p*_^2^ = 0.05, *p* = .44) and interaction of these factors (*F*(3, 48) = 0.71, η_*p*_^2^ = 0.04, *p* = .55) were insignificant. Because the number of participants for this analysis is not big enough, it is a limitation of this study that we cannot directly draw statistical conclusions on this issue. However, even for the participant who took part only in Experiment 2, thresholds for all conditions were above 31 ms; there were no thresholds below 30 ms, which were obtained for the anger, fear, and joyful faces in Experiment 1. Because there was no sign of difference among the facial expression conditions in any participant groups, it is unlikely that the differences among the facial expression conditions were lost due to the practice effect in Experiment 2.

## Experiment 3

Based upon the results of Experiment 1 and of our earlier study (Kobayashi & Ichikawa, 2016), we propose that the temporal resolution of visual processing increases with the increase of the degree of emotional arousal. However, facial expressions that evoke any emotion, irrespective of the arousal level, might increase the temporal resolution of visual processing compared to the state prevailing when one views a neutral face. With Experiment 3, we examined whether the temporal resolution of visual processing increased with the increase of the emotional arousal degree when viewing a sad face, which would evoke a medium degree of arousal with less variance than that associated with the joyful face used in Experiment 1, as well as anger and neutral faces, which would respectively evoke high and low degrees of emotional arousal (Russell & Mehrabian, 1977). In Experiment 3, after measuring the temporal resolution of visual processing, observers rated the experienced arousal and valence while viewing each facial expression image.

### Methods

#### Observers

Observers were 20 naïve students: 5 women and 15 men (*M* and *SD* of age were respectively 21.95 and 1.94 years). All had normal or corrected-to-normal visual acuity. None had participated in Experiment 1 or 2. Before the experiments, observers provided written informed consent to participate, similar to that described for the other two experiments.

#### Stimuli

We used the same stimuli in Experiments 1 and 2 for anger and neutral facial expressions. Additionally, we used sad facial expressions of the same actors from the same ATR facial expressions database. The stimulus size was the same as those used for Experiments 1 and 2.

#### Procedure

The same procedure as that used for Experiment 1 was used, except that the duration of the color facial expression stimuli before desaturation of the image was reduced from 1,000 to 500 ms.

After finishing the temporal resolution task, observers assigned a rating for each image. After each image was presented for 500 ms, observers rated the extent of experienced arousal and valence using a bipolar seven-point scale.

### Results and Discussion

We conducted a one-way repeated measures ANOVA with the facial expression as a factor for the arousal and valence ratings (Table A3). The main effect of the facial expressions was significant for the arousal rating [*F*(2, 38) = 152.87, η_*p*_^2^ = 0.89, *p *< .0001] and for the valance rating [*F*(2, 38) = 27.58, η_*p*_^2^ = 0.59, *p *< .0001]. Shaffer's post hoc tests with Bonferroni correction for the arousal rating revealed that the ratings for the angry expressions were higher than those for the sad and neutral expressions and that the ratings for the sad expressions were higher than those of the neutral expressions. Regarding the valence rating, the ratings for neutral expressions were higher than those of angry and sad expressions. These results matched our expectations (Russell & Mehrabian, 1977).

Data of five observers whose FA rate exceeded 50% in any condition were excluded. The mean FA rate of the remaining 15 observers was 6.9% (*SD* = 0.08).

**Figure 5.**
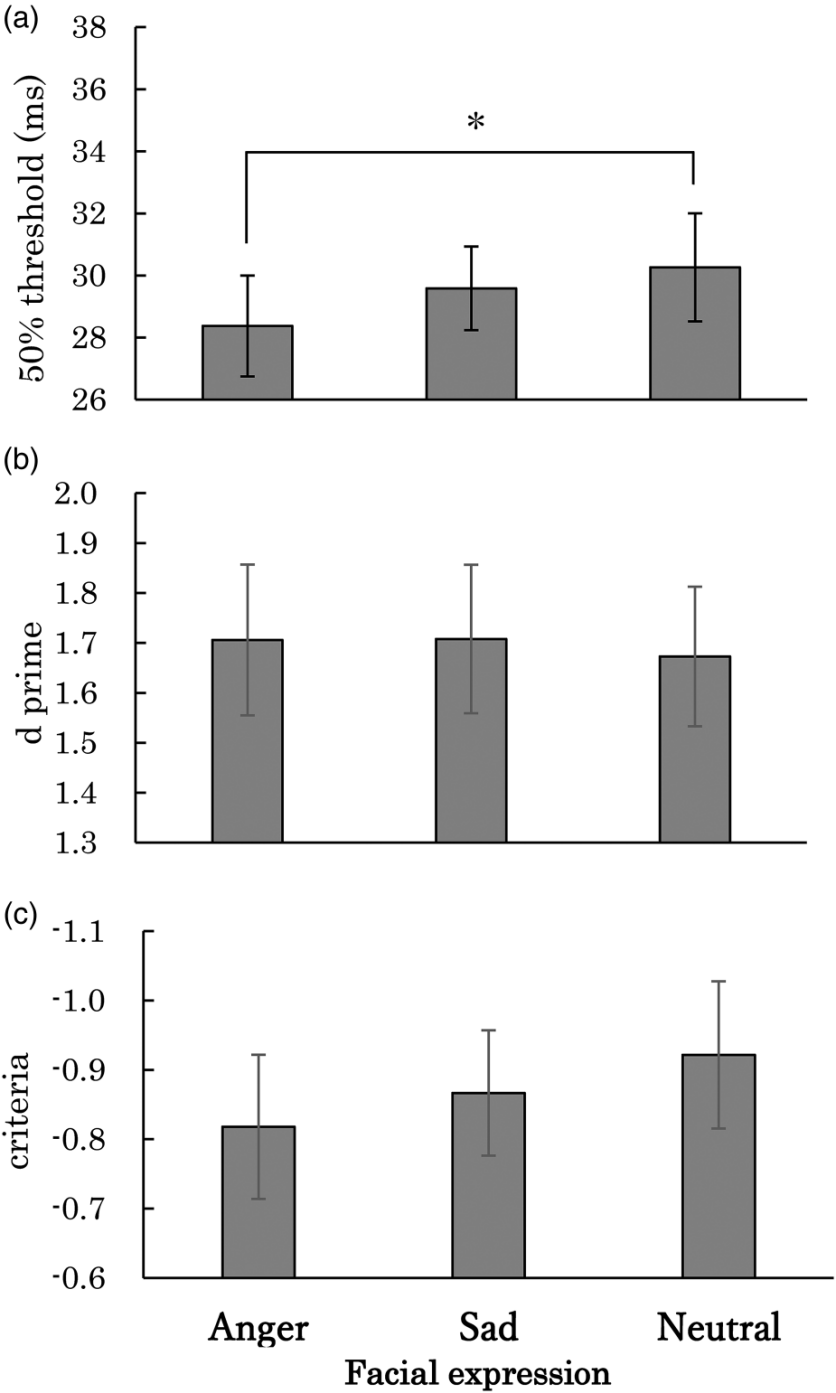
(a) Average of the 50% thresholds for the detection of desaturation for each facial expression condition in Experiment 3. (b) Average of *d*′ for each facial expression condition. (c) Average of the criteria for facial expression conditions. Error bars show SEM. *Note.* * means *p *< .05.

We conducted a Probit analysis to obtain individual 50% thresholds for the detection of desaturation for each condition. Figure 5 depicts the means of 50% thresholds for the respective facial expression conditions from 15 participants whose data fitting would be good (*p *> .05) in all the expression conditions in χ^2^ tests. The means of the upper-lower limits of the 95% confidence intervals with the estimated 50% thresholds for the anger, sad, and neutral conditions were 25.60–34.45 ms, 23.57–38.14 ms, and 26.83–36.81 ms, respectively. One-way repeated measures ANOVA with the facial expressions as a factor for the 50% thresholds found a significant main effect of facial expressions [*F*(2, 28) = 6.68, η_*p*_^2^ = 0.32, *p* = .0043]. Shaffer's post hoc tests with Bonferroni correction found that the threshold for the anger condition was lower than the threshold for the neutral condition (*p* = .0005). One-way Bayesian repeated measures ANOVA with facial expressions as a factor for the 50% thresholds for the detection of desaturation revealed a Bayes factor (BF_10_) of 9.431; post hoc tests revealed BF_10_ of 25.181 and 0.700, respectively, for the differences between the neutral face and anger and sad conditions. The value of the Bayes factor for the neutral and the angry face was within the level of the strong evidence for H_1_ and the value of the Bayes factor for the neutral and the sad face was, at most, at the level of the anecdotal evidence for H_1_.

We conducted a one-way repeated measures ANOVA with the facial expression as a factor for the *d′* and criteria. The analysis for both the *d′* and criteria found no significant main effect of emotion [*F*(2, 28) = 0.06, η_*p*_^2^ = 0.00, *p* = .9409 for *d′* and *F*(2, 28) = 1.31, η_*p*_^2^ = 0.09, *p* = .2858 for criteria]. These results show that the difference of 50% threshold among facial expressions cannot be attributed to the difference of criteria in detecting the desaturation of the pictures. One-way Bayesian repeated measures ANOVA with the facial expressions as a factor for the *d′* and criteria revealed Bayes factors (BF_10_) of 0.168 and 0.396, respectively. These values of the Bayes factors provide evidence in favor of the null hypothesis that no difference exists in performance between these facial expressions.

The correlation between the arousal rating for each picture and the 50% threshold for the detection of desaturation was found to be significant [*r* = −.68, *t*(10) = −2.93, *p* = .0149], whereas the correlation between the valence rating for each picture and the 50% threshold was found to be marginally significant [*r* = .53, *t*(10) = 1.98, *p* = .0758]. Also, correlation with *d′* was not significant with the arousal rating [*r* = .23, *t*(10) = −076, *p* = .4631] or with the valence rating [*r* = −.03, *t*(10) = −0.08, *p* = .9357]. These results suggest that the temporal resolution of visual processing increases along with the emotional arousal level evoked by viewing facial expressions, although the effects of valence on the temporal resolution of visual processing are unclear.

## General Discussion

Results of the present study revealed that viewing a fearful face increases the temporal resolution of visual processing of these images (Experiment 1) and confirmed that emotions evoked by viewing images of various facial expressions, not properties of images, influence the temporal resolution of visual processing using the face inversion effect (Experiment 2). The increased temporal resolution of visual processing was observed only for the upright version of the expressive facial images, but not for the inverted (upside down) version of the same image, although the properties of the images for the inverted version were identical to those of the upright version. The increased temporal resolution in visual processing reported for natural images in our earlier study (Kobayashi & Ichikawa, 2016) was contaminated by some properties of images such as color saturation, luminance, and spatial frequencies (Nomura & Yotsumoto, 2018). However, the results presented herein confirmed a highly increased temporal resolution in visual processing in terms of emotional response.

Results demonstrate that the 50% threshold for the detection of desaturation is lowered by viewing an emotional face. This result implies that the temporal sensitivity of visual processing is improved by an emotional response evoked by viewing facial expressions. Such effects of viewing facial expressions on visual temporal resolution are not restricted to the threshold of the detection of events in a short duration. As we described in the *Introduction*, Bocanegra and Zeelenberg (2011) reported that viewing a fearful face improves the temporal resolution of visual processing at a suprathreshold level. Actually, their three experiments demonstrated that the hit rates in the temporal discontinuity detection task after viewing low spatial frequency components of an upright face image were at least 0.650 for the neutral face condition and 0.846 for the fearful face condition. Their reports, regarded together with the results presented herein, indicate that the emotional response in terms of viewing a fearful face can improve the temporal resolution of visual processing at both the near-threshold level and at the suprathreshold level for detecting transient changes of the visual stimulus.

Results indicate that the temporal resolution of visual processing depends upon the emotional arousal evoked by viewing a facial expression, although the effects of valence on the temporal resolution of visual processing are not so clear in Experiments 1 and 3. In Experiment 3, the 50% threshold for the detection of desaturation, which is expected to correspond to the temporal resolution of visual processing, correlates with the rated arousal level for the facial expression images, although the correlation between the 50% threshold and rated valence was marginal. These results resemble those obtained from our earlier study (Kobayashi & Ichikawa, 2016): we found a significant correlation between the temporal resolution of visual processing and arousal scores of images selected from IAPS but not with their valence scores. The present results, considered together with the results of our earlier study, suggest that the degree of arousal is related to the increased temporal resolution of visual processing, irrespective of the category of emotions evoked by viewing images of facial expressions or natural scenes. The enhanced temporal resolution of visual processing that occurs when viewing dangerous scenes and fearful faces of companions, which would evoke strong arousal of emotion, is beneficial for survival in perilous situations that might produce those scenes and facial expressions. A mental state with high arousal would enable the visual system to be alerted and enable it to be ready to process the transient visual inputs to respond appropriately to those inputs.

The present study found only a marginal correlation between the temporal resolution of visual processing and the valence rating (Experiment 3). This result might derive from the small range of valence ratings among facial expression pictures used in the present study (mean valence ratings were from −1.94 to 1.59 and from −1.93 to −0.38, respectively, in Experiments 1 and 3), compared with the range of arousal ratings (mean arousal ratings were from −1.84 to 2.53 and from −1.45 to 2.45, respectively, in Experiments 1 and 3). Future studies should use a larger valence range to examine the effects of valence on the temporal resolution of visual processing.

Although the results of the present study are not useful to examine the anatomical underpinnings of these phenomena, we can speculate on the neural bases for the improvement of temporal resolution of visual processing. It has been suggested that emotional stimuli can be processed along two pathways: a slow and precise pathway and a fast and rough pathway (LeDoux, 1994). Along the fast and rough pathway, visual information would be processed in the superior colliculus and suprachiasmatic nucleus. It would reach the amygdala without processing in the visual cortex, which is involved in the recognition of the viewed objects. Bocanegra and Zeelenberg (2009) reported that emotion affects contrast sensitivity by influencing the visual processing of the magnocellular and parvocellular pathways in visual processing. The findings reported herein, together with those of our earlier study (Kobayashi & Ichikawa, 2016), suggest that this “slow” visual cortex can be speeded up in situations of high emotional arousal.

The present results suggest that the improvement of the temporal resolution of visual processing is associated with arousal. In fact, the increased temporal resolution of visual processing in terms of evoked emotional arousal has been reported not only from perilous situations but also from joyful actions. Sports experts often report that they visualize events as happening in slow motion when they concentrated intensely on their play. Such a mental state reported by experts is designated as “flow”. An individual within the flow state is regarded as in the “zone” (e.g., Csikszentmihalyi, 1990). A flow state, or getting into the zone, is often accompanied by the transformation of subjective time (slowing down, or sometimes, speeding up) and a joyful feeling, as well as effortless intense task-related attention, automatic action, a strong sense of control, and a reduced sense of external and internal awareness. Because joyful feelings that accompany with flow are characterized by strong positive valence emotion (e.g., Russell, 1980; Tseng et al., 2014), a person experiencing flow could be highly aroused. Slowing of the subjective time reported during the flow state might have a common underlying mechanism with the experience of slow motion in a dangerous situation, which might be related to the improvement of the temporal resolution in visual processing (examined in the present study). A high arousal mental state would enable visual processing to increase the temporal resolution of visual processing and would make a person visualize events in slow motion.

A recent report described that emotional arousal evoked by auditory stimulus facilitates visual attention in a visual search task (Asutay & Västfjäll, 2017). Another described that a fearful face image attracts visual attention (Phelps et al., 2006). Several studies have demonstrated that endogenous attention might improve the temporal resolution of visual processing (Hein et al., 2006; Shioiri et al., 2015), whereas exogenous attention, which would be elicited by a transient luminance change in a part of the visual field, degrades the temporal resolution of visual processing (Hein et al., 2006; Yeshurun & Levy, 2003). Based on the results of those studies, one might infer that visual attention, which is enhanced at a high arousal level, is involved in improving the temporal resolution of visual processing in viewing high arousal facial expressions. In addition, a psychophysics study demonstrated that the preparation of a ballistic reaching movement for a visual stimulus might increase the temporal resolution of visual processing and might also increase the perceived duration of a visual stimulus with no emotional response (Hagura et al., 2012). Results obtained from the present study imply that the emotional response is not necessary for increased temporal resolution of visual processing, although endogenous attention to a visual stimulus might be involved in the preparation of a reaching movement to the stimulus. These results of studies implicate endogenous attention as a key factor for increasing the temporal resolution of visual processing. Future studies should be undertaken to investigate whether emotional arousal by itself, visual attention enhanced through emotional arousal, or both increase the temporal resolution of visual processing.

The other important question is whether the effects of arousal are specific to the temporal resolution of visual processing. The possibility exists that arousal might improve performance in most cognitive tasks. In fact, Sharp et al. (2019) reported that endogenous attention might improve the performances of both a temporal segregation task, which would be related to the improvement of temporal resolution of visual processing, and a temporal integration task, which would be related to a decline in the temporal resolution. Emotional arousal would influence cognitive tasks of different types in a flexible manner, just as endogenous attention does. Future studies should be undertaken to examine the effects of the arousal response on the performance of different cognitive tasks.

## Appendix

See Tables A1–A3.

**Table A1.** Means of ratings for facial expressions, experienced arousal, and valences in Experiment 1.

		11-point scale (0–100)			7-point scale (from −3 to +3)	
Emotion	Person	Anger	Fear	Joy	Arousal	Valence
Anger	Female 1	**60.0**	24.7	5.3	2.2	−1.4
		**(2.78)**	(7.22)	(0.57)	(0.16)	(0.18)
	Female 2	**90.0**	25.3	4.2	2.5	−1.9
		**(5.95)**	(4.60)	(3.19)	(0.22)	(0.19)
	Male 1	**83.7**	21.1	1.6	2.7	−1.9
		**(5.66)**	(3.50)	(3.44)	(0.30)	(0.25)
	Male 2	**81.1**	26.8	1.1	2.8	−2.5
		**(4.14)**	(1.24)	(3.33)	(0.35)	(0.28)
Fear	Female 1	28.9	**64.2**	5.3	1.6	−1.5
		(7.68)	**(6.45)**	(2.74)	(0.17)	(0.20)
	Female 2	32.1	**61.6**	7.9	1.2	−1.7
		(5.55)	**(5.82)**	(2.49)	(0.15)	(0.20)
	Male 1	40.0	**60.0**	7.4	1.8	−1.6
		(7.40)	**(4.57)**	(2.75)	(0.28)	(0.32)
	Male 2	14.7	**81.6**	2.1	2.2	−1.8
		(3.00)	**(1.56)**	(2.51)	(0.36)	(0.24)
Joy	Female 1	5.3	2.6	**79.5**	0.2	1.8
		(3.62)	(5.80)	**(0.78)**	(0.13)	(0.20)
	Female 2	8.4	2.1	**73.7**	−0.6	1.5
		(6.59)	(5.59)	**(2.84)**	(0.18)	(0.20)
	Male 1	10.0	7.9	**84.7**	1.3	1.6
		(5.73)	(3.65)	**(1.81)**	(0.32)	(0.24)
	Male 2	2.1	1.6	**85.3**	0.4	1.4
		(3.07)	(4.14)	**(3.12)**	(0.36)	(0.42)
Neutral	Female 1	27.4	14.7	6.8	−1.8	−0.7
		(7.47)	(6.69)	(0.78)	(0.09)	(0.14)
	Female 2	13.7	7.4	14.7	−1.8	0.4
		(6.17)	(5.56)	(1.77)	(0.17)	(0.21)
	Male 1	16.8	8.4	4.2	−1.5	−0.8
		(4.79)	(2.18)	(1.32)	(0.28)	(0.15)
	Male 2	11.6	5.8	4.2	−2.2	−0.2
		(1.77)	(0.78)	(2.76)	(0.39)	(0.40)

Numbers in parentheses show SEM for the respective facial expressions. Rated values for the presented expressions are shown in bold typeface.

**Table A2.** Means of ratings for facial expressions, experienced arousal, and valences in Experiment 2.

		11-point scale (0–100)			7-point scale (from −3 to +3)	
Emotion	Person	Anger	Fear	Joy	Arousal	Valence
Anger	Female 1	**33.7**	24.2	36.8	1.2	−0.2
		**(7.46)**	(6.50)	(6.71)	(0.29)	(0.40)
	Female 2	**70.0**	33.2	3.7	2.3	−2.3
		**(7.37**	(6.54)	(1.57)	(0.32)	(0.20)
	Male 1	**72.6**	37.9	1.6	2.3	−2.3
		**(7.41)**	(6.78)	(0.86)	(0.35)	(0.23)
	Male 2	**75.8**	33.2	5.8	2.3	−1.9
		**(6.55)**	(6.76)	(3.77)	(0.37)	(0.36)
Fear	Female 1	22.6	**41.6**	12.1	0.9	−1.3
		(5.12)	**(6.03)**	(3.63)	(0.27)	(0.25)
	Female 2	22.6	**65.8**	4.7	1.3	−1.8
		(4.51)	**(6.23)**	(1.93)	(0.31)	(0.18)
	Male 1	22.1	**58.4**	13.7	1.9	−1.1
		(3.71)	**(5.42)**	(3.76)	(0.31)	(0.26)
	Male 2	26.3	**74.2**	12.1	1.9	−1.9
		(5.31)	**(6.50)**	(5.55)	(0.32)	(0.25)
Joy	Female 1	4.2	5.8	**68.9**	−0.5	1.7
		(1.92)	(3.36)	**(4.83)**	(0.35)	(0.24)
	Female 2	8.4	9.5	**62.6**	−0.2	1.7
		(3.61)	(4.49)	**(6.88)**	(0.34)	(0.28)
	Male 1	10.0	10.0	**72.6**	1.1	1.4
		(4.05)	(3.59)	**(6.53)**	(0.42)	(0.28)
	Male 2	6.3	7.4	**68.9**	0.1	1.5
		(2.44)	(4.18)	**(5.97)**	(0.36)	(0.28)
Neutral	Female 1	15.8	18.4	8.9	−1.9	−0.5
		(5.15)	(4.97)	(3.41)	(0.29)	(0.19)
	Female 2	14.7	8.4	17.9	−1.6	0.1
		(4.86)	(3.36)	(4.75)	(0.33)	(0.37)
	Male 1	17.9	12.6	7.4	−1.7	−0.3
		(4.75)	(4.25)	(2,74)	(0.32)	(0.17)
	Male 2	17.4	10.5	3.7	−1.7	−0.3
		(5.34)	(4.08)	(1.91)	(0.31)	(0.20)

Numbers in parentheses show SEM for each facial expression. Rated values for the presented expressions are shown in bold typeface.

**Table A3.** Means of ratings for experienced arousal and valence in Experiment 3.

	7-point scale (from −3 to +3)		
Emotion	Person	Arousal	Valence
Anger	Female 1	2.5	−1.9
		(0.13)	(0.26)
	Female 2	1.9	−1.7
		(0.27)	(0.29)
	Male 1	2.6	−2.2
		(0.15)	(0.26)
	Male 2	2.9	−2.0
		(0.08)	(0.34)
Sadness	Female 1	0.3	−1.4
		(0.28)	(0.22)
	Female 2	0.5	−1.9
		(0.30)	(0.22)
	Male 1	2.4	−1.8
		(0.16)	(0.22)
	Male 2	1.6	−1.1
		(0.13)	(0.24)
Neutral	Female 1	−1.6	0.5
		(0.33)	(0.29)
	Female 2	−1.3	−0.7
		(0.27)	(0.24)
	Male 1	−1.6	−0.5
		(0.29)	(0.21)
	Male 2	−1.4	−0.9
		(0.36)	(0.23)

Numbers in parentheses show SEM.
